# Dissecting the Association between Gut Microbiota and Brain Structure Change Rate: A Two-Sample Bidirectional Mendelian Randomization Study

**DOI:** 10.3390/nu15194227

**Published:** 2023-09-30

**Authors:** Huimei Huang, Shiqiang Cheng, Xuena Yang, Li Liu, Bolun Cheng, Peilin Meng, Chuyu Pan, Yan Wen, Yumeng Jia, Huan Liu, Feng Zhang

**Affiliations:** 1 Department of Nephrology, Xi’an Children’s Hospital, The Affiliated Children’s Hospital of Xi’an Jiaotong University, Xi’an 710003, China; 2 Key Laboratory of Trace Elements and Endemic Diseases of National Health and Family Planning Commission, School of Public Health, Health Science Center, Xi’an Jiaotong University, Xi’an 710061, China; chengsq0701@stu.xjtu.edu.cn (S.C.); smile940323@stu.xjtu.edu.cn (X.Y.); liuli0624@stu.xjtu.edu.cn (L.L.); boluncheng@xjtu.edu.cn (B.C.); mengpeilin@stu.xjtu.edu.cn (P.M.); ckecorn@stu.xjtu.edu.cn (C.P.); wenyan@mail.xjtu.edu.cn (Y.W.); jia.yu.meng@163.com (Y.J.); huan.liu@xjtu.edu.cn (H.L.)

**Keywords:** gut microbiota, brain structure, longitudinal changes, Mendelian randomization

## Abstract

The connection between the gut microbiota and brain structure changes is still unclear. We conducted a Mendelian randomization (MR) study to examine the bidirectional causality between the gut microbiota (211 taxa, including 131 genera, 35 families, 20 orders, 16 classes and 9 phyla; N = 18,340 individuals) and age-independent/dependent longitudinal changes in brain structure across the lifespan (N = 15,640 individuals aged 4~99 years). We identified causal associations between the gut microbiota and age-independent/dependent longitudinal changes in brain structure, such as family *Peptostreptococcaceae* with age-independent longitudinal changes of cortical gray matter (GM) volume and genus *Faecalibacterium* with age-independent average cortical thickness and cortical GM volume. Taking age-independent longitudinal changes in brain structure across the lifespan as exposures, there were causal relationships between the surface area and genus *Lachnospiraceae*. Our findings may serve as fundamentals for further research on the genetic mechanisms and biological treatment of complex traits and diseases associated with the gut microbiota and the brain structure change rate.

## 1. Introduction

The development of the human brain is a lengthy process that commences during the third week of gestation with the differentiation of neural progenitor cells and continues at least through late adolescence, and some argue, throughout the entire lifespan [1]. According to previous studies, this process is subject to the interplay of genetic factors and a dynamic environment [2]. Paus et al. established a correlation between the total brain volume and genetic variances within the KCTD8 locus among female adolescents [3]. They also observed a pronounced interaction between genes and the environment, particularly concerning the total cortical surface [3]. This observation was interpreted in the context of heightened adversity-induced apoptosis of progenitor cells during brain development, possibly influenced by a KCTD8 variant [3]. Socio-economic status, substance use, physical activity and nutrition are considered environmental factors that contribute to the development of the brain [4,5]. Longitudinal investigations play a pivotal role in pinpointing the genetic and environmental elements that impact the pace of alterations in the brain structure across the lifespan, encompassing both development and aging.

The gut microbiota, which co-develop with the host from birth, undergo dynamic changes throughout growth in response to various dietary patterns and pathological, physiological and environmental conditions [6]. Large-scale twin, family and population-based investigations have unveiled intriguing connections between the microbiome and host genetics, demonstrating that a proportion of bacterial taxa exhibits heritability [7,8,9,10]. Recently, metagenome-wide association studies (MWAS) have highlighted the potential roles of the gut microbiome in multiple complex conditions, such as neuropsychiatric disorders and autoimmune diseases, and they have delved into mechanistic explorations for diseases such as schizophrenia and obesity [11,12,13,14]. Cryan et al. proposed that the gut microbiota constitutes a central component of the signals within the microbiota–gut–brain axis, a bidirectional communication network involving the nervous system, gut microbiota, and neuroendocrine and neuroimmune pathways [15,16,17]. The gut microbiome has been proven to be associated with numerous physiological states, yet the debate regarding causality still hangs in doubt.

As the gut microbiome is considered to be involved in multiple complex traits and diseases and it interacts with the brain through the brain–gut axis, causality still remains an unresolved issue in this field. Mendelian randomization (MR) presents an opportunity to discern the causal and noncausal effects of exposures and outcomes based on cross-sectional data, obviating the need for randomized controlled trials or animal studies [18]. MR uses genetic polymorphisms as a proxy for exposure to infer a causal relationship between exposure and outcome. For example, a prior investigation employed MR to explore the relationships between ischemic heart disease and the gut microbiota [19]. More recently, MR was applied to validate that an elevated relative abundance of bacteria producing the fecal volatile short-chain fatty acid (SCFA) butyrate was causally linked to an improved insulin response to oral glucose challenges. Conversely, another fecal SCFA, propionate, was causally associated with an elevated risk of type 2 diabetes [20]. Nonetheless, the potential interactions between longitudinal changes in the brain structure over the lifespan and the gut microbiota were not clear until now.

In this study, by using the latest GWAS summary statistics of longitudinal lifespan brain structure changes and gut microbiota, we conducted a bidirectional MR analysis to systematically explore the interactions between longitudinal changes in brain structure across the lifespan and the gut microbiota. These findings may serve as fundamentals for further research on the genetic mechanisms and biological treatment of complex traits and diseases associated with the gut microbiota and brain structure changes.

## 2. Materials and Methods

### 2.1. GWAS Datasets of Longitudinal Lifespan Brain Structure Changes

The latest GWAS summary datasets of longitudinal lifespan brain structure changes scanned through magnetic resonance imaging on more than one occasion stem from a recently published investigation [2]. Briefly, Brouwer et al. conducted an extensive age-independent meta-analysis and age-dependent meta-regression GWAS analysis to pinpoint genetic loci associated with annual change rates in various morphological brain metrics. These encompassed eight global metrics (total brain excluding brainstem but including cerebellum, average cortical thickness, surface area measured at the gray–white matter boundary, volumes of the cortical and cerebellar gray and white matter, and total lateral ventricle volume) and seven subcortical metrics (caudate, thalamus, putamen, hippocampus, pallidum, nucleus accumbens and amygdala). This extensive analysis utilized data collected from 40 longitudinal cohorts, encompassing a total of 15,640 participants aged from 4 to 99 years. Given the nonuniform rate of brain structure changes across different ages [21] and the concurrent development and age-related shifts in gene expression [22], that study assessed whether the identified genetic variants exhibited age-dependent effects. In essence, this involved examining whether these variants differentially influenced the rates of brain changes at various life stages using genome-wide meta-regression models featuring linear or quadratic age effects. The basic characteristics of the study population, such as sample size and demographic information, are summarized in Supplementary Table S1.

### 2.2. GWAS Dataset of Gut Microbiota

The latest GWAS summary statistics of gut microbiota have been extracted from a recently conducted investigation [9]. Briefly, the researchers orchestrated the harmonization of 16S ribosomal RNA (rRNA) gene sequencing profiles and genotyping data from an extensive pool of 18,340 individuals collected from 24 different cohorts. The primary objective was to discern the influence of host genetics on the relative abundance and composition of the gut microbiota. Due to the included cohorts exhibiting differences in sample collection protocols, the DNA purification kits utilized for fecal sample DNA extraction, the genotyping array platforms, the specific 16S rRNA gene domains selected, the quality control steps after sequencing, and the software employed to merge paired-end sequencing tags, following rigorous quality control procedures and the merging of reads, all cohorts uniformly applied a standardized 16S processing pipeline (available at https://github.com/alexa-kur/miQTL_cookbook/) (accessed on 9 June 2022). This comprehensive analysis encompassed a total of 211 taxa, which included 9 phyla, 16 classes, 20 orders, 35 families and 131 genera. These taxa were retained for subsequent analysis as they met the established taxon inclusion criteria. For a more detailed account of the sample characteristics, quality control procedures, and statistical methodologies employed, please refer to the aforementioned earlier study. The basic characteristics of the study cohorts included in the GWAS of gut microbiota, such as sample size and demographic information, are summarized in Supplementary Table S2.

### 2.3. Assessing Bidirectional Causal Relationships between Longitudinal Lifespan Brain Structure Changes and Gut Microbiota

To evaluate the bidirectional causal relationship between brain structure changes and gut microbiota, we conducted a two-sample Mendelian randomization (MR) analysis employing the “TwoSampleMR” R package. We selected all single-nucleotide polymorphisms (SNPs) with a relatively lenient threshold of *p* < 1 × 10^−5^ in the MR analyses as instrument variables. This strategy allowed us to amass a larger number of SNPs for sensitivity analyses, an approach that has been widely adopted previously [20,23]. For instrumental variables, we exclusively retained independent SNPs characterized by an r^2^ value of less than 0.001 and situated within a 10,000 kb range, according to the 1000 Genomes European data implemented in the “TwoSampleMR” package. To gauge the robustness of the selected instrumental variables, we estimated the F statistic [24], with an F statistic exceeding 10 commonly considered as a typical threshold for strong instrumental variables [25]. We limited our analysis to results derived from at least three shared SNPs.

MR causality tests were assessed by using the Wald ratio, and we pooled Wald ratios through meta-analysis using the inverse variance weighted (IVW) method [26]. The IVW method operates on the assumption of no (unbalanced) horizontal pleiotropy. Additionally, we evaluated the causality using additional methods, such as the weighted median method [27], which served as an alternative approach to IVW. A nominal *p* value of 0.05 was used as the threshold for statistical significance, and in cases of the 211 endpoints for each of the rate of brain changes, we considered a *p* value below 2.37 × 10^−4^ (0.05/211) to be the multiple testing corrected level of significance. *p* values between 2.37 × 10^−4^ and 0.05 were considered to be suggestive of significance.

### 2.4. Sensitivity Analyses

The significant MR results were verified after correction by sensitivity analyses. First, we executed an MR-Egger regression to examine the potential bias of directional pleiotropy [28]. The intercept in the Egger regression indicates the mean pleiotropic effect of all genetic variants, which is interpreted as evidence of pleiotropy when the value differs from zero (*p* < 0.05). We further estimated the heterogeneity of our findings by employing Cochran’s Q statistic [26] and conducting leave-one-out analyses [29] to check whether the causal association was obviously driven by a single SNP (a *p* value of <0.05 was regarded as an outlier).

## 3. Results

**Figure 1:**
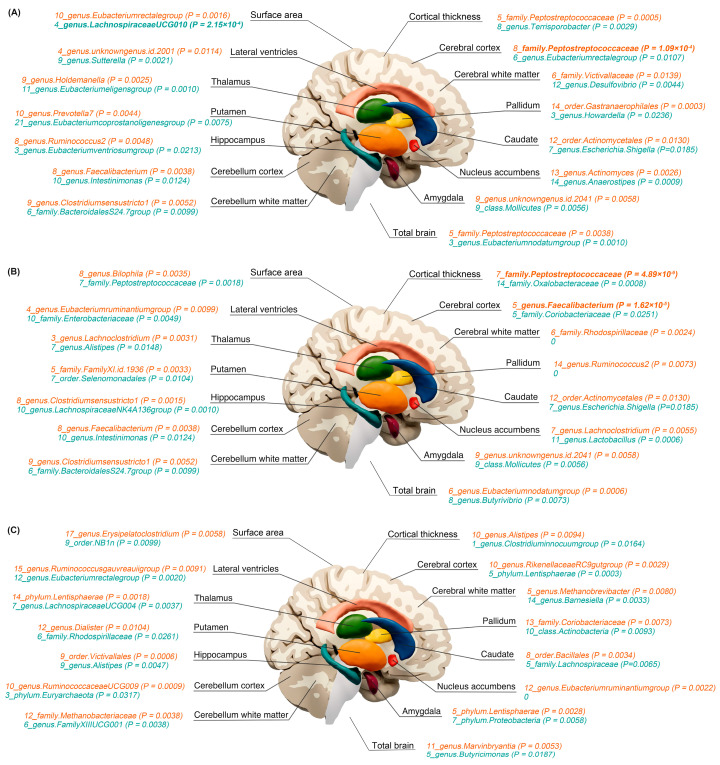
The most significant bidirectional causal associations between the gut microbiota and longitudinal lifespan brain structure changes. (**A**) Age−independent longitudinal lifespan brain structure changes as exposures or outcomes. (**B**) Age−dependent longitudinal linear lifespan brain structure changes as exposures or outcomes. (**C**) Age-dependent longitudinal quadratic lifespan brain structure changes as exposures or outcomes. All associations are presented as the number of significant/suggestive significant gut microbiota and brain structure pairs and the *p* values of the Mendelian randomization analysis. The text in orange indicates the gut microbiota as exposures and blue indicates the change rate of brain structures as exposures. The significant causal relationships are highlighted in bold face.

The most significant bidirectional causal associations between the gut microbiota and longitudinal lifespan brain structure changes are presented in Figure 1.

### 3.1. Causal Effect of Gut Microbiota on Longitudinal Lifespan Brain Structure Changes

With the age-independent longitudinal lifespan brain structure changes as outcomes, the IVW approach identified 1 significant and 130 suggestive causal relationships between the gut microbiota and the brain changes, such as family *Peptostreptococcaceae* with cortical gray matter (GM) volume (beta = 824.12, 95% confidence interval (CI) = 406.578~1241.66, *p* value = 1.09 × 10^−4^). The full results are summarized in Supplementary Table S3.

With the age-dependent longitudinal lifespan brain structure changes as outcomes, the IVW approach identified 2 significant and 109 suggestive causal relationships between the gut microbiota and the linear change rate of brain structures, such as genus *Faecalibacterium* with average cortical thickness (beta = −0.45, 95% CI = −0.64~−0.26, *p* value = 4.89 × 10^−6^) and cortical GM volume (beta = −95.90, 95% CI = −139.49~−52.31, *p* value = 1.62 × 10^−5^). In addition, the IVW approach identified 163 suggestive causal relationships between the gut microbiota and the quadratic change rate of brain structures, such as order *Victivallales* with hippocampus (beta = 0.04, 95% CI = 0.02~0.06, *p* value = 6.00 × 10^−4^) and genus *Ruminococcaceae* with cerebellum GM (beta = 0.39, 95% CI = 0.16~0.61, *p* value = 9.33 × 10^−4^). The full results are summarized in Supplementary Tables S4 and S5.

### 3.2. Causal Effect of Longitudinal Lifespan Brain Structure Changes on Gut Microbiota

With the age-independent longitudinal lifespan brain structure changes as exposures, the IVW approach identified 1 significant and 126 suggestive causal relationships between the brain changes and gut microbiota, such as surface area with genus *Lachnospiraceae* (beta = 6.41 × 10^−4^, 95% CI = −0.0010~−0.0003, *p* value = 2.15 × 10^−4^). The full results are summarized in Supplementary Table S6.

For the age-dependent longitudinal lifespan brain structure changes, the IVW approach identified 113 suggestive causal relationships between the gut microbiota and the linear change rate of brain structures, such as the nucleus accumbens with genus *Lactobacillus* (beta = −0.60, 95% CI = −0.94~−0.26, *p* value = 6.07 × 10^−4^) and average cortical thickness with family *Oxalobacteraceae* (beta = 0.56, 95% CI = 0.23~0.89, *p* value = 8.14 × 10^−4^). In addition, the IVW approach identified 99 suggestive causal relationships between the gut microbiota and the quadratic change rate of brain structures, such as cortical GM volume with phylum *Lentisphaerae* (beta = 0.06, 95% CI = 0.03~0.09, *p* value = 2.90 × 10^−4^) and class *Lentisphaeria* (beta = 0.06, 95% CI = 0.02~0.09, *p* value = 4.51 × 10^−4^). The full results are summarized in Supplementary Tables S7 and S8.

### 3.3. Bidirectional Causal Effects between Gut Microbiota and Longitudinal Lifespan Brain Structure Changes

We also observed bidirectional suggestive causal effects between the gut microbiota and longitudinal lifespan brain structure changes, including 8 pairs for the age-independent change rate, such as genus *Bifidobacterium* with the nucleus accumbens (beta_exposure_ = −3.67, 95% CI_exposure_ = −7.01~−0.32, P_exposure_ = 3.16 × 10^−2^; beta_outcome_ = 0.01, 95% CI_outcome_ = 0.003~0.017, P_outcome_ = 7.40 × 10^−3^), 2 pairs for the age-dependent linear change rate, such as genus *Lactobacillus* with the nucleus accumbens (beta_exposure_ = 0.13, 95% CI_exposure_ = 0.006~0.258, P_exposure_ = 3.99 × 10^−2^; beta_outcome_ = −0.60, 95% CI_outcome_ = −0.94~−0.26, P_outcome_ = 6.10 × 10^−4^), and 10 pairs for the age-dependent quadratic change rate of the brain structure, such as genus *Alistipes* with hippocampus (beta_exposure_ = −0.04, 95% CI_exposure_ = −0.067~−0.007, P_exposure_ = 1.58 × 10^−2^; beta_outcome_ = 1.00, 95% CI_outcome_ = 0.31~1.69, P_outcome_ = 4.67 × 10^−3^) (Table 1).

### 3.4. Sensitivity Analyses

We conducted a series of sensitivity analyses to corroborate the putative causal relationships between the gut microbiota and longitudinal lifespan brain structure changes obtained from bidirectional MR. First, leave-one-out analyses revealed that no single SNP influenced the causal estimates. The detailed results of the leave-one-out sensitivity analysis are presented in Supplementary Tables S9–S14. Second, the MR-Egger intercepts of all associations were found in close proximity to zero, suggesting the absence of significant pleiotropy. Third, the directions of the association from other MR methods were the same as those of the IVW method, which supports the reliability of our inferred causal effects. Overall, the sensitivity analyses confirmed the reliability of our putative causal effects in both the forward and reverse MR results.

## 4. Discussion

This bidirectional MR analysis provides evidence in favor of interactions between the gut microbiota and age-independent and age-dependent longitudinal lifespan brain structure changes. To the best of our knowledge, this is the first large-scale MR study to systematically identify the bidirectional causal relationship between the gut microbiota and change rate of brain structure across the lifespan. As reported by previous studies, the gut microbiota and altered brain growth or rates of decline are implicated in multiple complex diseases, such as autoimmune diseases, neuropsychiatric disorders and metabolic diseases [12,14,30]. Our findings may serve as a foundation for further research on the genetic mechanisms and biological treatment of those complex diseases.

We observed a causal effect of family *Peptostreptococcaceae* on cortical gray matter (GM) volume. The family of *Peptostreptococcaceae*, belonging to the order *Clostridiales*, comprises several genera, namely *Acetoanaerobium*, *Peptostreptococcus*, *Filifactor*, *Sporacetigenium*, *Proteocatella* and *Tepidibacter* [31]. Previous studies have reported that *Peptostreptococcaceae* were dominant in healthy controls compared with insomnia patients and significantly decreased in a depression rat model [32,33]. Interestingly, recent findings have shown that the cortical gray matter structure has the capacity to predict the subsequent onset of depression, and certain cortical and subcortical grey matter regions have been linked to the severity of insomnia [34,35]. Based on this evidence, we hypothesized that *Peptostreptococcaceae* could potentially play a mediating role in the communication between gut microbiota and the brain during the development of depression and insomnia disorders. However, further studies are needed to validate this hypothesis.

Genus *Faecalibacterium* showed a causal effect on the average cortical thickness and cortical GM volume in the current study. In a recent randomized clinical trial, the impact of a 5-week treatment involving high-frequency and low-frequency deep transcranial magnetic stimulation (dTMS), as well as sham stimulation, on the gut microbiota composition of individuals with obesity was investigated. Interestingly, the high-frequency dTMS group exhibited a significant increase in the abundance of *Faecalibacterium* reads compared to their baseline levels [36]. Given the capacity of dTMS for modulating cortical excitability, the reward system and, indirectly, the autonomic nervous system [37], the researchers proposed the hypothesis that dTMS might influence the brain–gut communication pathways and, consequently, the composition of the gut microbiota in individuals with obesity [36]. However, further experimental studies are warranted to explore potential associations between microbiota changes and metabolic and neurohormonal alterations.

We found that surface area had a significant causal effect on genus *Lachnospiraceae* in our MR analysis. *Lachnospiraceae*, the main genera detected in human intestine, can be detected in early infancy and it is even present in meconium [38]. It has been reported by a recent study that preterm infants with suboptimal head circumference growth, an established early marker for neurodevelopment outcomes, exhibited a reduction in the abundance or prevalence of *Lachnospiraceae* [39]. In addition, both cortical surface area and *Lachnospiraceae* appeared to be involved in depressive syndromes and other neuropsychiatric disorders [40,41,42]. Oliphant et al. demonstrated that neonatal systemic inflammation in rats can result in changes in blood–brain barrier permeability and behavior. Therefore, it is plausible that *Lachnospiraceae* may impact neurodevelopment by influencing energy resources and immune responses [39]. Given that human brain development is a protracted process commencing in the third gestational week [1], we propose that cortical surface area may mediate depression and other neuropsychiatric disorders through its potential effects on *Lachnospiraceae.*

We also observed bidirectional suggestive causal effects between the gut microbiota and longitudinal lifespan brain structure changes through the MR analysis. The ‘gut–microbiota–brain axis’ encompasses a complex network of interactions between various biological systems, enabling bidirectional communication between gut bacteria and the brain. This axis plays a vital role in maintaining the balance of the host’s gastrointestinal, central nervous and microbial systems [43]. As reported by a previous study, the gut microbiota has been implicated in numerous conditions, including anxiety, autism, schizophrenia, Alzheimer’s disease and Parkinson’s disease [43]. Future studies aimed at comprehending the mechanisms involving the microbiota–gut–brain axis and exploring microbial-based interventions and therapeutic strategies for these intricate disorders are of paramount interest.

According to previous studies, the relative abundance of the *Actinobacteria* phylum was linked to magnetic resonance imaging–diffusion tensor imaging variable differences in the thalamus, hypothalamus and amygdala between obese and non-obese subjects [44]. In schizophrenia patients, the regional homogeneity indexes in the right superior temporal cortex and the left cuneus were negatively correlated with the abundance of the genus *Roseburia* [45]. However, we found that there is limited reported information about the brain structure and gut microbiota from healthy humans and the available data from human and rodent studies do not directly address the associations between specific brain parameters and the gut microbiota we examined in our study. We believe that emphasizing the current gaps in this area will underscore the need for future investigations.

MR is a powerful tool for inferring causality from genetic data, but it does come with its own set of assumptions and limitations. One of the key assumptions in MR analysis is the validity of instrumental variables. Instrumental variables should be strongly associated with the exposure of interest, independent of confounders, and unrelated to the outcome except through the exposure. It is crucial to mention that violations of these assumptions can potentially bias MR estimates. In addition, genetic variants may affect the outcome through pathways other than the exposure. This is an important consideration because it can introduce bias into MR estimates.

However, some limitations of the current study should be noted. Firstly, we only observed a few significant bidirectional causal relationships between gut microbiota and age-independent and age-dependent longitudinal lifespan brain structure changes. In accordance with established guidelines for conducting MR analyses, adopting an excessively conservative approach to multiple testing is deemed unnecessary. This is due to the generally limited statistical power of MR studies and the inherent nature of MR, which typically explores exposure–outcome relationships with pre-existing epidemiological or biological support [46]. We suggest that the suggestive causal associations observed between the gut microbiota and the change rate of the brain structure should also be given attention. Secondly, it is noteworthy that the GWAS summary statistics utilized in the current study predominantly originate from European populations. Therefore, it is essential to exercise caution when generalizing the findings to other ethnic groups. Thirdly, the primary objective of this study was to evaluate the bidirectional causal relationships between gut microbiota and age-independent or age-dependent longitudinal lifespan brain structure changes. To corroborate these findings and elucidate the potential genetic mechanisms underpinning the observed interactions, further functional experimental research is warranted. Moreover, potential confounders, including diet, lifestyle and medication use may influence the observed associations between the gut microbiota and changes in brain structure. These traits have been reported by previous studies to influence the gut microbiota and alterations of brain structure. Thus, the importance of conducting more longitudinal studies cannot be overstated, as they are essential for identifying the genetic and environmental factors that influence alterations in the abundance and composition of gut microbiota throughout the course of development and aging.

## 5. Conclusions

In summary, by adopting the widely used genetic approach, we performed a large-scale two-sample bidirectional MR analysis to explore the causal associations between the gut microbiota and age-independent and age-dependent longitudinal lifespan brain structure changes. Our study identified modest interactions between the gut microbiota and age-independent and age-dependent longitudinal lifespan brain structure changes. These findings may provide novel ideas for future research on the pathogenesis of complex traits and diseases associated with the gut microbiota and change rate of brain structures.

## Figures and Tables

**Table 1:** The significant bidirectional causal associations between the gut microbiota and longitudinal lifespan brain structure changes.

Exposures	Outcomes	No. of SNPs	Method	Beta (95% CI)	*P*	Heterogeneity Test		Pleiotropy Test
						Cochran’s Q	*P*	*P*_Intercept_
*Family* *Peptostreptococcaceae*	Age-independent cortical GM volume	10	IVW	824.12 (406.58~1241.66)	1.09 × 10^−4^	9.73	0.37	/
		10	WM	602.15 (5.48~1198.82)	4.79 × 10^−2^	/	/	/
		10	MR Egger	−1237.93 (−3192.64~716.78)	0.25	/	/	0.07
*Genus Faecalibacterium*	Linear change rate of average cortical thickness	5	IVW	−0.45 (−0.64~−0.26)	4.89 × 10^−6^	0.92	0.92	/
		5	WM	−0.47 (−0.73~−0.21)	3.82 × 10^−4^	/	/	/
		5	MR Egger	−0.17 (−0.98~0.64)	0.71	/	/	0.53
*Genus* *Faecalibacterium*	Linear change rate of cortical GM volume	5	IVW	−95.90 (−139.49~−52.31)	1.62 × 10^−5^	0.91	0.92	/
		5	WM	−94.5804 (−154.88~−34.28)	2.11 × 10^−3^	/	/	/
		5	MR Egger	−68.3635 (−250.71~113.98)	0.52	/	/	0.78
Age-independent surface area	*Genus* *Lachnospiraceae*	4	IVW	6.41 × 10^−4^ (−0.001~−0.0003)	2.15 × 10^−4^	2.26	0.52	/
		4	WM	6.19 × 10^−4^ (−0.001~−0.0002)	5.17 × 10^−3^	/	/	/
		4	MR Egger	3.20 × 10^−5^ (−0.004~0.004)	0.99	/	/	0.77

GM, gray matter; CI, confidence interval; SNPs, single nucleotide polymorphisms; IVW, inverse variance weighted; WM, weighted median.

## Data Availability

All of the GWAS summary datasets included in the current study were obtained with written informed consent from the participants and were approved by ethics committees. No further ethical consent was required since this study is based on publicly available summary-level data. The gut microbiota GWAS summary statistics were extracted from the tables in the main manuscript or the Supplementary Materials of the original publication and from the repository (https://mibiogen.gcc.rug.nl/) (accessed on 17 April 2022). Regarding the brain structure change rate, the GWAS summary-level data are available on the ENIGMA consortium webpage (http://enigma.ini.usc.edu/research/download-enigma-gwas-results) (accessed on 17 April 2022).

## Supplementary Materials

The following supporting information can be downloaded from: https://www.mdpi.com/article/10.3390/nu15194227/s1, Table S1: Basic characteristics of the individuals included in the GWAS of longitudinal lifespan brain structure changes; Table S2: Basic characteristics of the individuals included in the GWAS of gut microbiota; Table S3: The causal effect of gut microbiota on age-independent longitudinal changes in brain structure across the lifespan; Table S4: The causal effect of gut microbiota on age-dependent linear change rate of brain structure across the lifespan; Table S5: The causal effect of gut microbiota on age-dependent quadratic change rate of brain structure across the lifespan; Table S6: The causal effect of age-independent longitudinal changes in brain structure across the lifespan on gut microbiota; Table S7: The causal effect of age-dependent linear change rate of brain structure across the lifespan on gut microbiota; Table S8: The causal effect of age-dependent quadratic change rate of brain structure across the lifespan on gut microbiota; Table S9: The leave-one-out sensitivity analysis of gut microbiota on age-independent longitudinal changes in brain structure across the lifespans; Table S10: The leave-one-out sensitivity analysis of gut microbiota on age-dependent linear change rate of brain structure across the lifespan; Table S11: The leave-one-out sensitivity analysis of gut microbiota on age-dependent quadratic change rate of brain structure across the lifespan; Table S12: The leave-one-out sensitivity analysis of age-independent longitudinal changes in brain structure across the lifespan on gut microbiota; Table S13: The leave-one-out sensitivity analysis of age-dependent linear change rate of brain structure across the lifespan on gut microbiota; Table S14: The leave-one-out sensitivity analysis of age-dependent quadratic change rate of brain structure across the lifespan on gut microbiota.
